# Optimized PCR conditions minimizing the formation of chimeric DNA molecules from MPRA plasmid libraries

**DOI:** 10.1186/s12864-019-5847-2

**Published:** 2019-07-11

**Authors:** Evgeniya S. Omelina, Anton V. Ivankin, Anna E. Letiagina, Alexey V. Pindyurin

**Affiliations:** 1 Institute of Molecular and Cellular Biology SB RAS, Novosibirsk, Russia; 2 Novosibirsk State University, Novosibirsk, Russia

**Keywords:** Massively parallel reporter assay (MPRA), Emulsion PCR (ePCR), Conventional PCR, Chimeric DNA molecules, Next-generation sequencing, Barcode

## Abstract

**Background:**

Massively parallel reporter assays (MPRAs) enable high-throughput functional evaluation of various DNA regulatory elements and their mutant variants. The assays are based on construction of highly diverse plasmid libraries containing two variable fragments, a region of interest (a sequence under study; ROI) and a barcode (BC) used to uniquely tag each ROI, which are separated by a constant spacer sequence. The sequences of BC–ROI combinations present in the libraries may be either known a priori or not. In the latter case, it is necessary to identify these combinations before performing functional experiments. Typically, this is done by PCR amplification of the BC–ROI regions with flanking primers, followed by next-generation sequencing (NGS) of the products. However, chimeric DNA molecules formed on templates with identical spacer fragment during the amplification process may substantially hamper the identification of genuine BC–ROI combinations, and as a result lower the performance of the assays.

**Results:**

To identify settings that minimize formation of chimeric products we tested a number of PCR amplification parameters, such as conventional and emulsion types of PCR, one- or two-round amplification strategies, amount of DNA template, number of PCR cycles, and the duration of the extension step. Using specific MPRA libraries as templates, we found that the two-round amplification of the BC–ROI regions with a very low initial template amount, an elongated extension step, and a specific number of PCR cycles result in as low as 0.30 and 0.32% of chimeric products for emulsion and conventional PCR approaches, respectively.

**Conclusions:**

We have identified PCR parameters that ensure synthesis of specific (non-chimeric) products from highly diverse MPRA plasmid libraries. In addition, we found that there is a negligible difference in performance of emulsion and conventional PCR approaches performed with the identified settings.

**Electronic supplementary material:**

The online version of this article (10.1186/s12864-019-5847-2) contains supplementary material, which is available to authorized users.

## Background

Massively parallel reporter assays (MPRAs) allow high-throughput functional analysis of different DNA regulatory elements (e.g. enhancers and promoters) or their mutant variants [1–3]. Briefly, a set of DNA sequences to assay are cloned in a plasmid vector outside of a reporter gene coding sequence, and the resulting MPRA library, which typically contains a high number of plasmid variants, is used to transfect the cells of interest. To trace each individual DNA sequence under study, each molecule in the library is uniquely marked by a short (from a few to up to several dozen bp in length) barcode (BC), which is present within an untranslated region of the reporter gene [4–12]. Thus, the influence of each DNA sequence on the reporter expression can be measured by counting of the associated BC in the reporter RNA-seq data.

**Fig. 1:**
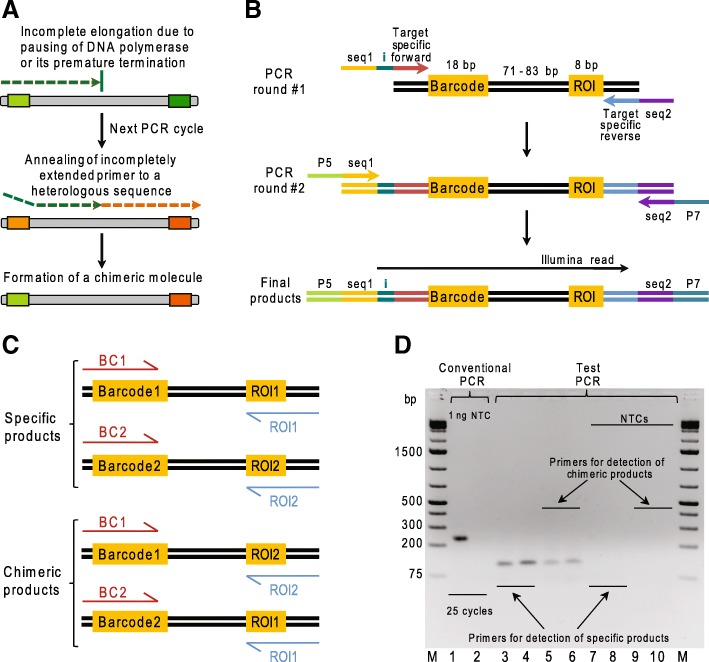
Formation of chimeric products during PCR using two-plasmid template and test PCR for their detection. **a** A scheme of formation of chimeric DNA molecules. **b** Amplification of BC–ROI fragments from plasmid templates by two consecutive rounds of PCR. Products of round #2 PCR contain all necessary elements for Illumina NGS: sequences necessary for attachment of amplicons to a flow cell surface (“P5” and “P7”), sequencing primer sites (“seq1” and “seq2”) and a custom 8-bp sample index sequence (“i”). “Target specific forward” and “target specific reverse” denote sequences immediately flanking BC and ROI. Black horizontal arrow indicates a fragment of PCR products analyzed by NGS in this study. **c** Structures of all possible PCR products for the two-plasmid template system. Red and blue horizontal arrows indicate positions of specific primers used for the test PCR. **d** Agarose gel electrophoresis analysis of PCR products. Conventional PCR products were generated using either 1 ng of an equimolar mixture of plasmid#1 and plasmid#2 (lane 1) or water (no template control, “NTC”; lane 2) as template and primers Libr-A_1_-for/Libr-rev. Test PCR were set up using either ^1^/_100_th of the purified products of conventional one-round PCR (lanes 3–6) or water (no template controls, “NTCs”; lanes 7–10) as template and the following primers: BC1/ROI1 (lanes 3 and 7), BC2/ROI2 (lanes 4 and 8), BC1/ROI2 (lanes 5 and 9) and BC2/ROI1 (lanes 6 and 10). M stands for GeneRuler 1 kb Plus DNA Ladder (Thermo Fisher Scientific)

Importantly, the sequences of BCs and/or the associated DNA regions of interest (hereafter ROIs) in MPRA plasmid libraries are frequently not known a priori, as random oligonucleotides are used to clone these elements [5, 10, 13]. To identify all unique BC–ROI combinations, PCR amplification followed by the next-generation sequencing (NGS) is typically used. However, it has been previously shown that routine conventional PCR co-amplification of DNA sequences containing two variable motifs (in the case of MPRAs, BC and ROI) separated by a constant region frequently leads to formation of undesired chimeric molecules, from 5.4 to 30% [14–22]. Such PCR products complicate and can mislead the MPRA data analysis as well as decrease the productivity of the approach, as the association of the same BC with different ROIs leads to the elimination of all such BCs and ROIs from the analysis. Chimeric DNA molecules seem to result from the annealing of incompletely extended primers to a heterologous target sequence during PCR [23]. Incompletely elongated DNA strands are presumably the consequence of the pausing of DNA polymerase on the template, or of its premature termination. As a result, mixed-template amplification leads to the formation of chimeric PCR products, which are composed of two artificially combined sequences [24] (Fig. 1a). The frequency of chimeric molecule formation appears to be a function of the length and sequence similarity of the co-amplified DNA molecules [17]. Additionally, it is known that the amount of the DNA template, the number of amplification cycles, the size of plasmid library, and duration of the extension step play crucial roles in the formation of chimeric PCR products [25, 26]. However, the presence of different DNA template molecules within the same PCR reaction mixture is thought to be the main reason for chimeric molecule formation [14, 27]. Thus, the emulsion PCR (ePCR) method, which provides a simple physical separation of the template DNA molecules due to the usage of water-in-oil emulsion [28], seems to be a solution of the problem.

Here, we first describe an optimized two-round protocol for ePCR amplification of the BC–ROI region of MPRA constructs, which minimizes formation of chimeric products. To develop the protocol, an equimolar mixture of two plasmids containing unique 18-bp BC and 8-bp ROI sequences separated by constant 71-bp region was used as a template. By adjusting ePCR settings (amount of template, number of amplification cycles and duration of the extension step), we were able to reduce the frequency of the chimeric PCR products arisen between the assayed BC–ROI regions to only 0.22%. Next, we applied the same conditions of ePCR for amplification of two high-diversity BC–ROI plasmid libraries (each containing > 30,000 of unique clones). NGS of these samples demonstrated that on average they contain 0.30% of spurious BC–ROI combinations. Finally, we surprisingly found that performing conventional PCR in the same optimized conditions results in an almost identical proportion of chimeric products (on average 0.32%) from the plasmid libraries. Thus, we conclude that the optimized conventional PCR is the simplest and most cost-effective approach to amplify a priori unknown combinations of BCs and ROIs present in MPRA plasmid libraries for their subsequent identification by NGS.

## Results

### Chimeric molecule formation during conventional PCR using two-plasmid template

To assess the frequency of chimeric product formation during PCR, we first designed a simple system consisting of two very similar plasmid constructs, each containing unique 18-bp BC and 8-bp ROI sequences separated by a constant 71-bp region (hereafter plasmid#1 and plasmid#2). The plasmids were mixed at an equal molar ratio and used as templates in a one-round conventional PCR to amplify 236-bp BC–ROI fragments (Fig. 1b). To detect the presence of chimeric BC–ROI combinations in these PCR products, we designed the BC1, BC2, ROI1 and ROI2 primers, specific for the BCs and ROIs of plasmid#1 and plasmid#2 (Fig. 1c) and used BC1/ROI2 and BC2/ROI1 pairs in a test PCR. To exclude the formation of chimeric products during this test PCR, we adjusted the parameters of the analysis using a mixture of plasmid#1 and plasmid#2 templates. Namely, 12 amplification cycles at the annealing temperature of 60 °C, but not 14 amplification cycles at 55 °C, resulted in the absence of false positives (Additional file 1: Figure S1). Therefore, the former conditions were used for all subsequent test PCR runs. The analysis of the products of the one-round conventional PCR demonstrated that they include detectable amounts of chimeric BC–ROI molecules (Fig. 1d).

### Optimized two-round ePCR suppresses formation of the chimeric molecules from two-plasmid template

**Fig. 2:**
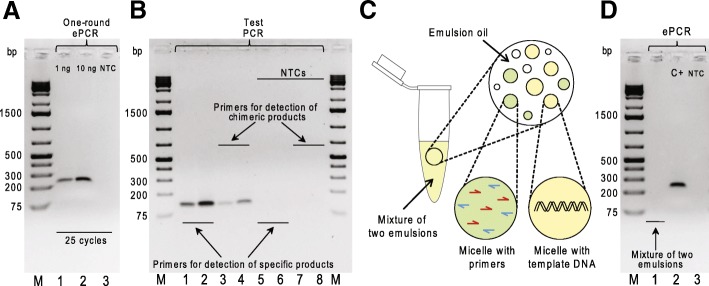
One-round ePCR does not suppress formation of the chimeric molecules from two-plasmid template. **a** Agarose gel electrophoresis analysis of products of one-round ePCR generated using either the indicated amounts of an equimolar mixture of plasmid#1 and plasmid#2 (lanes 1–2) or water (no template control, “NTC”; lane 3) as template and primers Libr-A_1_-for/Libr-rev. **b** Agarose gel electrophoresis analysis of products of test PCR set up using either ^1^/_100_th of the purified “1 ng” one-round ePCR sample (lanes 1–4) or water (no template controls, “NTCs”; lanes 5–8) as template and the following primers: BC1/ROI1 (lanes 1 and 5), BC2/ROI2 (lanes 2 and 6), BC1/ROI2 (lanes 3 and 7) and BC2/ROI1 (lanes 4 and 8). **c** The principle of the water-in-oil emulsion stability assay. Emulsion PCR is set up using a mixture of two emulsions that contain micelles lacking primers or template DNA. In stable emulsions, micelles do not merge together that results in no amplification products. **d** Agarose gel electrophoresis analysis of ePCR products generated using either a mix of micelles lacking primers Libr-A_1_-for/Libr-rev or plasmid#1 DNA (lane 1) or micelles with all the reaction components (positive control, “C+”; lane 2) or micelles with water instead of the template DNA (no template control, “NTC”; lane 3). M stands for GeneRuler 1 kb Plus DNA Ladder (Thermo Fisher Scientific) in (**a**, **b**, **d**)

We next amplified the BC–ROI fragments using ePCR. We utilized the Micellula DNA Emulsion & Purification Kit and mostly followed the manufacturer’s recommendations about the ePCR parameters (micelle count, amount of DNA template, PCR buffer composition in reaction tubes, PCR product purification, etc.). Specifically, we prepared 50-μl reaction mixtures containing 10^9^–10^10^ micelles and 2 × 10^8^ or 2 × 10^9^ plasmid molecules (1 ng or 10 ng of 4349-bp long plasmid#1 and plasmid#2 mixed at a molar ratio of 1:1). After 25 cycles of one-round ePCR, the emulsions were broken with 2-butanol and the products were purified. Agarose gel electrophoresis confirmed the presence of the expected 236-bp DNA fragments in the “1 ng” and “10 ng” ePCR samples (Fig. 2a). However, a test PCR on the purified ePCR products, even obtained starting with 1 ng of the plasmid#1/plasmid#2 template, detected chimeric BC–ROI molecules (Fig. 2b).

It is known that even traces of detergent may cause emulsion instability, i.e. cause a merge of individual micelles. Since for ePCR we used the Phusion DNA polymerase together with Phusion HF Buffer that is not detergent-free, we checked whether the stability of the emulsion was compromised. We mixed up two emulsions for ePCR: an emulsion containing the primers without the DNA template, and a second emulsion with only the template and no primers (Fig. 2c). If mixed emulsions are unstable, then the micelles would merge leading to the synthesis of PCR products. However, we did not observe any products from ePCR performed on this emulsion mixture (Fig. 2d), suggesting that other factors are responsible for generation of chimeric BC–ROI molecules, likely too many template DNA molecules or too many amplification cycles.

**Fig. 3:**
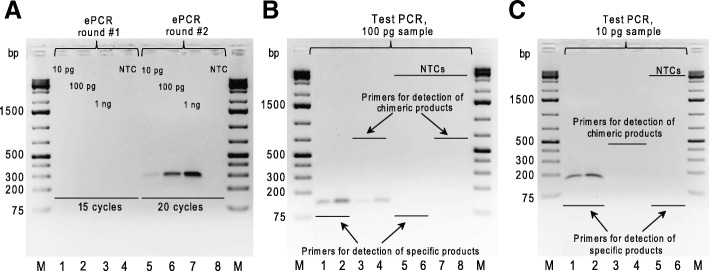
Optimized two-round ePCR effectively prevents formation of the chimeric molecules from two-plasmid template. **a** Agarose gel electrophoresis analysis of round #1 and round #2 ePCR products. Round #1 ePCR samples were generated using either the indicated amounts of an equimolar mixture of plasmid#1 and plasmid#2 (lanes 1–3) or water (no template control, “NTC”; lane 4) as template and primers Libr-A_1_-for/Libr-rev. Round #2 ePCR products were obtained using either ^1^/_100_th of the purified round #1 ePCR samples (lanes 5–7) or water (no template control, “NTC”; lane 8) as template and primers Libr-P5-for/Libr-P7-rev. **b** Agarose gel electrophoresis analysis of products of test PCR set up using either ^1^/_100_th of the purified “100 pg” round #2 ePCR sample (lanes 1–4) or water (no template controls, “NTCs”; lanes 5–8) as template and the following primers: BC1/ROI1 (lanes 1 and 5), BC2/ROI2 (lanes 2 and 6), BC1/ROI2 (lanes 3 and 7) and BC2/ROI1 (lanes 4 and 8). **c** Agarose gel electrophoresis analysis of products of test PCR set up using either ^1^/_100_th of the purified “10 pg” round #2 ePCR sample (lanes 1–4) or water (no template controls, “NTCs”; lanes 5–6) as template and the following primers: BC1/ROI1 (lanes 1 and 5), BC2/ROI2 (lanes 2 and 6), BC1/ROI2 (lane 3) and BC2/ROI1 (lane 4). M stands for GeneRuler 1 kb Plus DNA Ladder (Thermo Fisher Scientific) in (**a**, **b**, **d**)

To reduce formation of chimeric BC–ROI molecules in ePCR, we decreased the initial amount of template DNA and performed the amplification with two subsequent rounds (Fig. 1b) of 15 and 20 cycles, as single reactions with a higher number of cycles may exhaust resources of individual micelles. Fifty μl reactions of round #1 ePCR contained approximately 10^9^–10^10^ micelles and 2 × 10^6^, 2 × 10^7^ or 2 × 10^8^ plasmid molecules (10 pg, 100 pg or 1 ng of 4349-bp long plasmid#1 and plasmid#2 mixed at a molar ratio of 1:1). After 15 cycles of round #1 ePCR, the emulsions were broken, and the products were purified and analyzed by agarose gel electrophoresis. No bands were observed in the gel (Fig. 3a), most likely due to low amounts of the DNA template coupled with the low number of amplification cycles. Next, we set up 50-μl reactions of round #2 ePCR containing approximately 10^9^–10^10^micelles and ^1^/_100_th (0.5 μl) of the purified ePCR products of round #1. After 20 cycles of round #2 ePCR, we broke the emulsions and purified the products from “10 pg”, “100 pg” and “1 ng” template mixtures. Agarose gel electrophoresis revealed a clear direct relationship between the amounts of the 289-bp DNA fragments and the number of template DNA molecules used for round #1 ePCR (Fig. 3a). Notably, a test PCR performed on the purified “10 pg”, but not on “100 pg”, of round #2 ePCR products did not detect chimeric BC–ROI molecules (Fig. 3b). Thus, we conclude that the initial amount of the DNA templates should be ~ 10^3^–10^4^-fold lower than the estimated total count of micelles in the emulsion, so as to minimize the chance of inclusion of two or more template molecules in the same micelle and prevent the formation of chimeric products.

To accurately measure the proportion of chimeric BC–ROI molecules present in “10 pg” round #2 ePCR products we subjected them to Illumina NGS analysis, which revealed on average 1.51% of chimeric products (Table 1). Next, we tried to further reduce the proportion of chimeric products by an additional optimization of the ePCR conditions. First, the amount of the DNA template used in round #2 ePCR was reduced from 0.5 μl to 0.3 μl. Second, the number of amplification cycles in round #2 ePCR was decreased from 20 to 18. NGS analysis of the amplification products showed that both conditions substantially reduce the proportion of chimeric BC–ROI molecules (Table 1). Specifically, round #2 ePCR samples obtained with reduced DNA template and diminished amplification cycles contained on average 0.66 and 0.22% of spurious products, respectively. Taken together, our findings indicate that synthesis of chimeric DNA molecules during amplification of BC–ROI fragments from a mixture of two different templates can be suppressed to almost negligible levels using two-round ePCR with optimized parameters.

**Table 1:** Occurrence of chimeric BC–ROI combinations under different ePCR conditions for the two-plasmid template system

Round #2 ePCR conditions	Total read count				Reads with chimeric BC–ROI combinations, %					
	R#1		R#2		BC1–ROI2		BC2–ROI1		Average	
	BC1	BC2	BC1	BC2	R#1	R#2	R#1	R#2	R#1	R#2
0.5 μl of round #1 ePCR products, 20 cycles	134,011	134,779	47,072	46,333	1.579	1.651	1.465	1.347	1.522	1.499
0.3 μl of round #1 ePCR products, 20 cycles	43,782	44,788	10,327	10,452	0.831	0.629	0.737	0.440	0.784	0.535
0.5 μl of round #1 ePCR products, 18 cycles	21,042	21,733	166,862	170,934	0.200	0.225	0.230	0.211	0.215	0.218

The settings of the round #1 ePCR were the same for all samples: 15 amplification cycles starting with ~ 2 × 10^6^ template DNA molecules

R#1 and R#2 stand for replicate 1 and replicate 2, respectively

### Application of the two-round PCR approach for amplification of BC–ROI fragments of MPRA libraries

When only two plasmid variants are co-amplified, the frequency of chimeric molecule formation may be underestimated because some micelles can host identical template molecules (this is primarily true for micelles with two plasmid templates). However, during ePCR co-amplification of a large number of homologous DNA sequences the probability of inclusion of identical template molecules in the same micelle is extremely low. Therefore, the proportion of chimeric molecules in multi-template ePCR samples may be higher than that detected for the two-plasmid template system described above. To check that, we applied the two-round ePCR approach for amplification of BC–ROI fragments from a couple of high diverse MRPA libraries, library-71 and library-83. Plasmids of both libraries contain 18-bp BC and 8-bp ROI sequences synthesized using degenerate oligonucleotides (and therefore not known a priori), which are separated by a constant 71-bp (library-71) or 83-bp (library-83) spacer (Fig. 1b). First, we amplified BC–ROI regions of library-71 using the optimal ePCR parameters defined for the two-plasmid template system (10 pg of template DNA in round #1 ePCR, 0.5 μl of the purified products of round #1 ePCR as template and 18 amplification cycles in round #2 ePCR). Based on the NGS data obtained, we defined a set of genuine BC sequences present in the plasmid library as described previously [29]. Next, we analyzed ROI sequences associated with each genuine BC and considered a sequence present in more than half of reads as a genuine one. Reads with the same genuine BC but other ROI sequences were regarded as chimeric ePCR products (for details, see Methods). We detected the formation of chimeric BC–ROI molecules with the average frequency of 0.57%, which is more than two-fold higher than that determined for the two-plasmid system (Table 2, ePCR, elongation time of 10 s). To further optimize ePCR conditions, we increased the duration of the extension step from 10 to 30 s. With this modified ePCR parameter, we amplified the BC–ROI regions of library-71 and library-83 and subjected the products to NGS. The analysis of sequencing data indicated a high reproducibility of the measurements both between replicates and libraries, and that on average the formation of chimeric products was decreased almost 2-fold, down to 0.30% (Table 2, ePCR, elongation time of 30 s).

**Table 2:** Occurrence of chimeric BC–ROI combinations under different PCR conditions for high-diversity MPRA libraries

Type of PCR	Elongation time, s^a^	MPRA library		Total read count		Reads with chimeric BC–ROI combinations, %	
		name	size^b^	R#1	R#2	R#1	R#2
emulsion	10	library-71	22,621	526,056	497,217	0.580	0.555
	30	library-71	51,650	594,511	514,941	0.342	0.310
	30	library-83	38,156	371,889	456,380	0.261	0.304
conventional	30	library-71	85,249	776,878	784,138	0.336	0.337
	30	library-83	52,654	788,801	809,402	0.295	0.310

^a^Indicated elongation time was used in both rounds of PCR

^b^The number of genuine BCs found in both replicates

R#1 and R#2 stand for replicate 1 and replicate 2, respectively

**Fig. 4:**
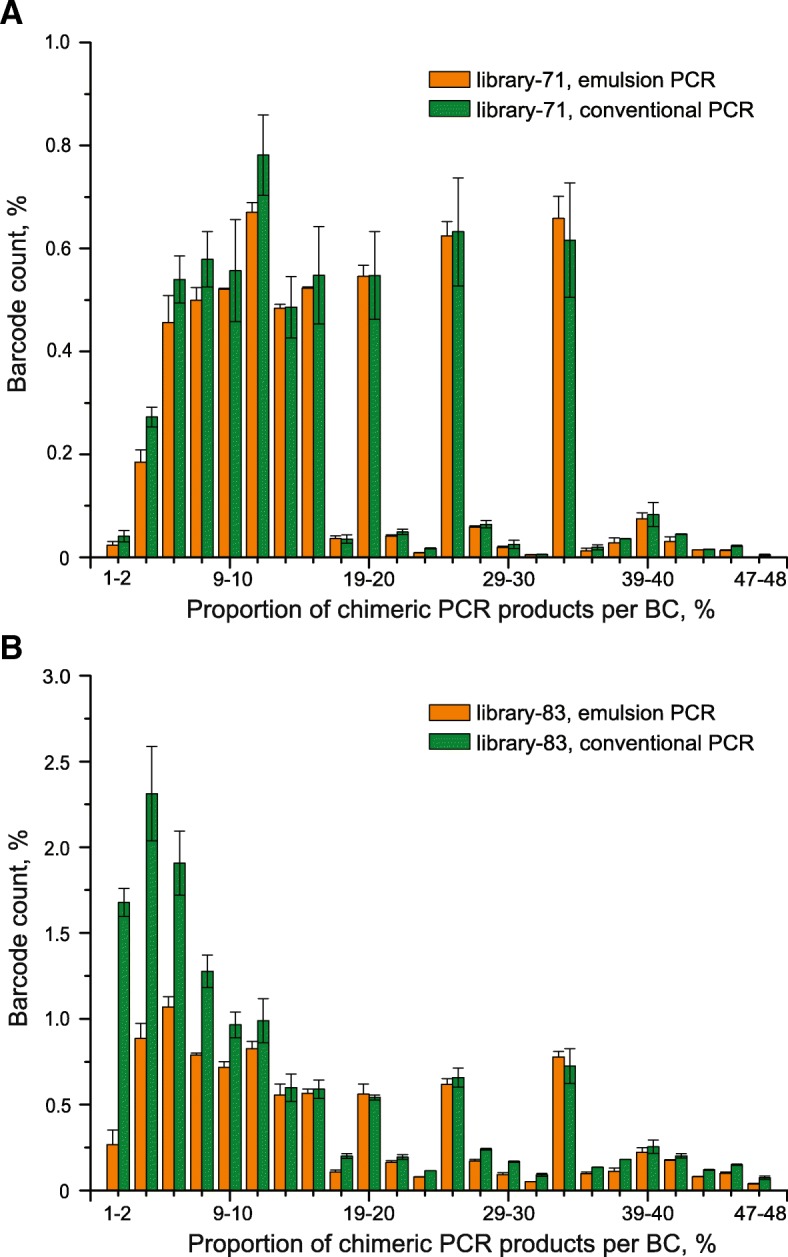
Comparison of PCR approaches in terms of proportion of chimeric products generated per BC from MPRA libraries. BC–ROI regions of library-71 and library-83 were amplified by emulsion and conventional two-round PCR using the same settings (10 pg of template DNA, 15 amplification cycles and elongation time of 30 s in round #1 PCR, ^1^/_100_th of the purified products of round #1 PCR as template, 18 amplification cycles and elongation time of 30 s in round #2 PCR) and subsequently subjected to NGS. The experiments were done in duplicates and averaged values of proportion of chimeric products per BC are plotted as histograms separately for library-71 (**a**) and library-83 (**b)**. Pronounced peaks at middle values of proportion of chimeric products per BC are mainly a result of low NGS coverage of some BCs (i.e., the peaks primarily represent cases with 1 chimeric BC–ROI combination per several genuine ones)

Next, to directly compare the performance of emulsion and conventional PCR approaches, we repeated the amplification of BC–ROI regions of both plasmid libraries using conventional PCR with exactly the same settings used for ePCR (10 pg of template DNA in round #1 PCR, 0.5 μl of the purified products of round #1 PCR as template and 18 amplification cycles in round #2 PCR, elongation time of 30 s in both rounds of PCR). Unexpectedly, NGS analysis showed that the proportion of chimeric BC–ROI molecules in conventional PCR products (0.32% on average) is just slightly higher than that observed for ePCR products (Table 2, conventional PCR, elongation time of 30 s). Moreover, the analysis of the chimeric molecule frequencies per BC showed that conventional PCR leads to a slight increase of low-abundant spurious products compared with ePCR, which was more pronounced for library-83 (Fig. 4). However, this did not affect the average frequency of chimeric products (reads) in samples prepared by ePCR and conventional PCR. Thus, we conclude that both, emulsion and conventional PCR approaches with the optimized settings can be successfully used for effective identification of initially unknown BC–ROI associations present in MPRA plasmid libraries.

## Discussion

It is well known that co-amplification of a large number of very similar sequences, such as 16S rRNA genes, may result in recombination events and the formation of chimeric DNA molecules [30–34]. Although multiple studies analyzed the effects of the sequence similarity, elongation time, number of amplification cycles, different types of DNA polymerases and initial template concentration on the proportion of chimeric PCR products [15–17, 19, 25, 35], the issue is still relevant, especially for high-throughput functional studies such as MPRAs, in which two beforehand unknown DNA sequences are separated by a constant region.

In this study, we optimized sample preparation using emulsion and conventional PCR approaches for the subsequent NGS-based identification of combinations of variable DNA sequences present in diverse MPRA plasmid libraries. First, using a simple two-plasmid template system, we found parameters of two-round ePCR that resulted in as low as 0.22% of chimeric products. However, amplification reactions performed with exactly the same settings on an MPRA library resulted in about 2.6-fold increase in the proportion of chimeras. This increase is most likely caused by the different frequencies with which the individual micelles of the two systems include two different template molecules. In the multi-template system, most double inclusions will involve different molecules that can give rise to chimeric PCR products, whereas in the two-plasmid system one half of the double inclusions will be identical molecules that are unable to produce chimeras. To optimize the amplification parameters, we also increased 3-fold the duration of the extension step in order to maximize the chances for incompletely elongated DNA strands to complete the synthesis. Although, the Phusion High-Fidelity DNA Polymerase used in the reactions can synthesize DNA strands of 300–700 nucleotides in 10 s, and the total length of the amplified regions was below 300 bp (thus, they all can be expected to be synthesized in 10 s), an increase of the extension step from 10 to 30 s led to a pronounced (1.9-fold) decrease of the proportion of chimeric products. Thus, to minimize the formation of chimeric PCR products the duration of the extension step should be substantially increased over the standard recommended/used times.

Recently, it has been reported that the ePCR method reduces the formation of chimeric DNA molecules by a factor of 38 compared with conventional PCR (1.5% vs. 57%) [27]. However, we have surprisingly found that under optimized conditions conventional PCR performs almost as effectively as ePCR. We suggest that the differences between these two PCR methods are eliminated by the modification of parameters; namely, low initial amount of DNA template (approximately 2 × 10^6^ molecules), two-round amplification, number of PCR cycles, and duration of the extension step. Since conventional PCR is much less laborious than ePCR, it can be recommended for the preparation of samples for NGS-based identification of a priori unknown sequence combinations present in MPRA libraries. Finally, we note that further adjustment of PCR settings might be required depending on the specific structure of the plasmid libraries.

## Conclusions

The phenomenon of chimeric PCR product formation complicates and can potentially distort the results of functional MPRA assays relying on high-diversity plasmid libraries containing two random DNA sequences separated by a known constant region. In this study, using specific MPRA libraries as templates, we defined a number of PCR parameters that substantially suppress the formation of the chimeras. Importantly, both emulsion and conventional PCR performed with our optimized parameters result in almost the same low proportions (less than 0.5%) of chimeric DNA molecules in the amplified samples.

## Methods

### Conventional PCR

#### One-round conventional PCR

The 50-μl PCR mixtures contained 1 ng of equimolar mixture of plasmid#1 and plasmid#2 as template, 1 × Phusion HF Buffer (Thermo Fisher Scientific), 200 μM dNTPs, 0.5 μM primers Libr-A_1_-for/Libr-rev (primer sequences are provided in Table 3) and 2 U of Phusion High-Fidelity DNA Polymerase (Thermo Fisher Scientific). Amplification was performed under the following conditions: 95 °C for 1 min; 25 cycles of 95 °C for 10 s, 55 °C for 30 s, and 72 °C for 10 s; 72 °C for 5 min. Aliquots of 10 μl of the reactions were analyzed by agarose gel electrophoresis.

**Table 3:** Oligonucleotide primers used in the study

Primer name	Sequence 5′ → 3′	Notes
Libr-A_1_-for	TCGTCGGCAGCGTCAGATGTGTATAAGAGACAG **TTCGGAGT** *GACACTCGAGGATCGAG*	Illumina sequencing primer sites “seq1” and “seq2” are underlined by single and double lines, respectively.
Libr-A_2_-for	TCGTCGGCAGCGTCAGATGTGTATAAGAGACAG **ACTCATTT** *GACACTCGAGGATCGAG*	
Libr-A_3_-for	TCGTCGGCAGCGTCAGATGTGTATAAGAGACAG **GGGATCCG** *GACACTCGAGGATCGAG*	
		The 8-bp sample index (“i”) sequences are in bold.
Libr-A_5_-for	TCGTCGGCAGCGTCAGATGTGTATAAGAGACAG **CAAGATAA** *GACACTCGAGGATCGAG*	
		“Target specific forward” and “target specific reverse” sequences immediately flanking BC and ROI are in italic.
Libr-A_6_-for	TCGTCGGCAGCGTCAGATGTGTATAAGAGACAG **GGACAACG** *GACACTCGAGGATCGAG*	
Libr-A_7_-for	TCGTCGGCAGCGTCAGATGTGTATAAGAGACAG **AGCGAGCT** *GACACTCGAGGATCGAG*	
Libr-A_8_-for	TCGTCGGCAGCGTCAGATGTGTATAAGAGACAG **CTGCACGT** *GACACTCGAGGATCGAG*	
Libr-A_9_-for	TCGTCGGCAGCGTCAGATGTGTATAAGAGACAG **GCACTAGT** *GACACTCGAGGATCGAG*	
Libr-rev	GTCTCGTGGGCTCGGAGATGTGTATAAGAGACAG *CCCTAGAAAGATAATCATATTGT*	
Libr-P5-for	AATGATACGGCGACCACCGAGATCTACACTCGTCGGCAGCGTC	Illumina “P5” and “P7” adapters are underlined by single and double lines, respectively.
Libr-P7-rev	CAAGCAGAAGACGGCATACGAGATGTCTCGTGGGCTCGG	
BC1	ACTAGGCAAGGCACCAG	
BC2	CGAGGGATATAGAGCGAGTTAA	
ROI1	TACGTTAAAGATAATCATGCATAAGTCG	
ROI2	TACGTTAAAGATAATCATGCTATATGGC	
Illumina-qPCR-1	AATGATACGGCGACCACCGA	KAPA Library Quantification Kit (Roche).
Illumina-qPCR-2	CAAGCAGAAGACGGCATACGA	

#### Two-round conventional PCR

Round #1 PCR mixtures (50 μl) contained 10 pg of MPRA library-71 or library-83 as template, 1 × Phusion HF Buffer (Thermo Fisher Scientific), 200 μM dNTPs, 0.5 μM primers Libr-A_N_-for/Libr-rev (Table 3), 0.5 mg/ml BSA (EUR_X_) and 2 U of Phusion High-Fidelity DNA Polymerase (Thermo Fisher Scientific). Amplification was performed under the following conditions: 95 °C for 1 min; 15 cycles of 95 °C for 10 s, 55 °C for 30 s, and 72 °C for 30 s; 72 °C for 5 min. The PCR products were column-purified with the GeneJET PCR Purification Kit (Thermo Fisher Scientific) and eluted in 50 μl of nuclease-free water.

Round #2 PCR mixtures (50 μl) contained 0.5 μl of the purified round #1 PCR products as template, 1 × Phusion HF Buffer (Thermo Fisher Scientific), 200 μM dNTPs, 0.5 μM primers Libr-P5-for/Libr-P7-rev (Table 3), 0.5 mg/ml BSA (EUR_X_) and 2 U of Phusion High-Fidelity DNA Polymerase (Thermo Fisher Scientific). Amplification was performed under the following conditions: 95 °C for 1 min; 18 cycles of 95 °C for 10 s, 52 °C for 30 s, and 72 °C for 30 s; 72 °C for 5 min.

### Emulsion PCR

Emulsions were prepared and the products were purified according to the instructions of the Micellula DNA Emulsion & Purification Kit (EUR_X_). An oil-surfactantmixture was freshly assembled, mixed thoroughly by vortexing and precooled to 4 °C. Water phase samples were mixed on ice as described below. Emulsion PCR products were column-purified after breaking the emulsions by addition of 1 ml of 2-butanol; DNA was eluted in 50 μl of the kit elution buffer preheated to 65 °C.

#### One-round ePCR

The 50-μl ePCR mixtures contained either 1 ng or 10 ng of equimolar mixture of plasmid#1 and plasmid#2 as template, 1 × Phusion HF Buffer (Thermo Fisher Scientific), 200 μM dNTPs, 0.5 μM primers Libr-A_1_-for/Libr-rev (Table 3), 0.5 mg/ml BSA (EUR_X_) and 2 U of Phusion High-Fidelity DNA Polymerase (Thermo Fisher Scientific). Amplification was performed under the following conditions: 95 °C for 1 min; 25 cycles of 95 °C for 10 s, 55 °C for 30 s, and 72 °C for 10 s; 72 °C for 5 min. Aliquots of 10 μl of the purified reaction products were analyzed by agarose gel electrophoresis.

#### Two-round ePCR

Round #1 ePCR mixtures (50 μl) contained 10 pg, 100 pg or 1 ng of equimolar mixture of plasmid#1 and plasmid#2 or 10 pg of MPRA library-71 or library-83 as template, 1 × Phusion HF Buffer (Thermo Fisher Scientific), 200 μM dNTPs, 0.5 μM primers Libr-A_N_-for/Libr-rev (Table 3), 0.5 mg/ml BSA (EUR_X_) and 2 U of Phusion High-Fidelity DNA Polymerase (Thermo Fisher Scientific). Amplification was performed under the following conditions: 95 °C for 1 min; 15 cycles of 95 °C for 10 s, 55 °C for 30 s, and 72 °C for 10 or 30 s (for details, see Results); 72 °C for 5 min. Aliquots of 10 μl of the purified reaction products were analyzed by agarose gel electrophoresis.

Round #2 ePCR mixtures (50 μl) contained 0.3 or 0.5 μl of the purified round #1 ePCR products as template (for details, see Results), 1 × Phusion HF Buffer (Thermo Fisher Scientific), 200 μM dNTPs, 0.5 μM primers Libr-P5-for/Libr-P7-rev (Table 3), 0.5 mg/ml BSA (EUR_X_) and 2 U of Phusion High-Fidelity DNA Polymerase (Thermo Fisher Scientific). Amplification was performed under the following conditions: 95 °C for 1 min; 18 or 20 cycles (for details, see Results) of 95 °C for 10 s, 52 °C for 30 s, and 72 °C for 10 or 30 s (for details, see Results); 72 °C for 5 min. Aliquots of 10 μl of the purified reaction products were analyzed by agarose gel electrophoresis.

### Test PCR for the detection of the chimeric amplification products

The 50-μl PCR mixtures contained 0.5 μl of the purified amplification products as template, 1 × DreamTaq buffer (Thermo Fisher Scientific), 200 μM dNTPs, 0.5 μM primers BC1/ROI1 or BC2/ROI2 or BC1/ROI2 or BC2/ROI1 (Table 3) and 2.5 U of Taq DNA polymerase (Thermo Fisher Scientific). Amplification was performed under the following conditions: 95 °C for 1 min; 12 or 14 cycles (for details, see Results) of 95 °C for 10 s, 55 or 60 °C (for details, see Results) for 15 s, and 72 °C for 10 s; 72 °C for 5 min. Aliquots of 10 μl of the reactions were analyzed by agarose gel electrophoresis.

### Quantitative real-time PCR

To accurately measure the concentration of DNA libraries for Illumina NGS, we used quantitative real-time PCR. The 20-μl PCR mixtures contained purified conventional or emulsion PCR round #2 products diluted 50, 100 or 200 times as template, 1 × BioMaster HS-qPCR SYBR Blue (Biolabmix) and 0.25 μM primers Illumina-qPCR-1 and Illumina-qPCR-2 (Table 3). Amplification was performed in a CFX96 Touch Real-Time PCR Detection System (Bio-Rad) under the following conditions: 95 °C for 5 min, 35 cycles of 95 °C for 30 s, 60 °C for 45 s. All measurements were done in three replicates.

### Illumina NGS and data analysis

Sequencing of 151 nucleotide-longsingle-end reads was performed on an Illumina MiSeq machine using MiSeq Reagent Kit v3 150 cycles (Illumina). Fastq files were processed and the data were analyzed using custom-made scripts (for details, see Additional file 2, Additional file 3: Table S1). Briefly, BC and ROI sequences were extracted from the reads. Next, a set of genuine BCs was defined as described previously [29]. Namely, at this step mutant versions of BCs (arisen due to PCR and/or NGS errors) that contain up to 2 nucleotide substitutions were identified and associated with the appropriate intact BCs. Only genuine BCs found in at least 2 reads were kept for the downstream analysis. Then, for each genuine BC the coupled ROI sequences were counted. The ROI sequence found in more than one half of the reads carrying the genuine BC (with Frequency > 0.5) was considered as genuine ROI. ROI sequences differing by 1 nucleotide from the genuine ROI were considered as variations arisen due to PCR and/or NGS errors, while all other ROI sequences were considered to be a result of chimeric PCR.

## Additional files

Additional file 1: **Figure S1.** Optimization of test PCR for detection of chimeric products synthesized during amplification of BC–ROI fragments from two-plasmid template. (PDF 132 kb)

Additional file 2: NGS data processing and analysis pipeline description. (PDF 175 kb)

Additional file 3: **Table S1.** Examples of identified BC–ROI combinations. (PDF 93 kb)

## Data Availability

The raw NGS datasets generated in the study and custom-made scripts for their analysis are not publicly available, but can be obtained upon request to the corresponding author.

## Abbreviations

BC: Barcode
ePCR: Emulsion PCR
MPRA: Massively parallel reporter assay
NGS: Next-generation sequencing
NTC: No template control
