# Supplementary material for: Optimized PCR conditions minimizing the formation of chimeric DNA molecules from MPRA plasmid libraries
Source: BMC Genomics. 2019 Jul 11;20(Suppl 7):536. doi: 10.1186/s12864-019-5847-2 (PMC6620194; doi:10.1186/s12864-019-5847-2)
Supplement: Supplementary file 1 — Figure S1. Optimization of test PCR for detection of chimeric products synthesized during amplification of BC–ROI fragments from two-plasmid template. (PDF 132 kb) [file 12864_2019_5847_MOESM1_ESM.pdf]

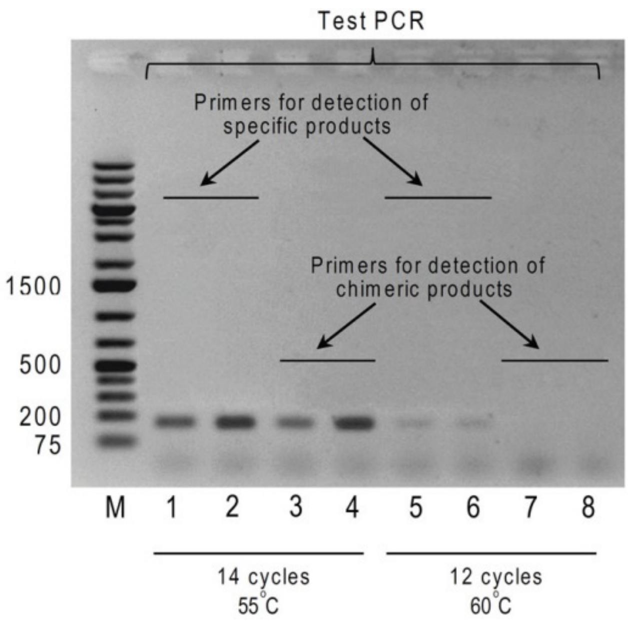

Figure S1. Optimization of test PCR for detection of chimeric products synthesized during amplification of BC–ROI fragments from two-plasmid template. Agarose gel electrophoresis analysis of test PCR products obtained at the indicated parameters using an equal molar mixture of plasmid#1 and plasmid#2 as template, and the following primers: BC1/ROI1 (lanes 1 and 5), BC2/ROI2 (lanes 2 and 6), BC1/ROI2 (lanes 3 and 7) and BC2/ROI1 (lanes 4 and 8). M stands for GeneRuler 1 kb Plus DNA Ladder (Thermo Fisher Scientific).
