# Supplementary material for: Optimized PCR conditions minimizing the formation of chimeric DNA molecules from MPRA plasmid libraries
Source: BMC Genomics. 2019 Jul 11;20(Suppl 7):536. doi: 10.1186/s12864-019-5847-2 (PMC6620194; doi:10.1186/s12864-019-5847-2)
Supplement: Supplementary file 2 — NGS data processing and analysis pipeline description. (PDF 175 kb) [file 12864_2019_5847_MOESM2_ESM.pdf]

NGS data processing and analysis pipeline description

1. **Structure of the reads.** All Illumina reads obtained in this study had the following structure: 8-nt NGS index, 17-nt constant part 1 (CP1), 18-nt BC, 71-nt or 83-nt constant part 2 (CP2)*, 8-nt region of interest (ROI), and 29-nt or 17-nt constant part 3 (CP3)*. Sequences of BCs and ROIs were not known *a priori*.
   *The CP2 and CP3 were of 71 and 29 nucleotides for the plasmid library-71 or 83 and 17 nucleotides for the plasmid library-83.

2. **Demultiplexing of fastq files.** Reads within each fastq file generated in this study were analyzed for the presence of the expected 8-nt NGS index sequences (no errors were allowed) at their beginnings. The exact sequences of the NGS indexes used are marked in bold within the sequences of Libr-A_N-for primers provided in **Table 3**. Reads with identified NGS indexes were split into separate subsets according to their index sequences. Then, the indexes were trimmed from the reads of each subset. In the subsequent steps of data analysis, each subset of reads (belonging to a separate experimental replicate) was analyzed independently as described below.

3. **Identification of BC and ROI sequences.** Reads were analyzed for the presence of (i) the 17-nt CP1 (1 error was allowed), (ii) 18-nt BC, (iii) 71-nt or 83-nt CP2 (see above; 7 and 8 errors were allowed, respectively), (iv) 8-nt ROI, (v) 29-nt or 17-nt CP3 (see above; 2 errors were allowed). The sequences of BC and ROI were not allowed to contain ambiguous nucleotides ("N" symbols). Only reads in which all these elements were identified were retained for the subsequent analysis.

4. **Identification of genuine BCs.** Based on the BC sequences extracted from the reads, a set of genuine BCs was defined using an algorithm described previously [1]. Namely, at this step mutant versions of BCs (arisen due to PCR and/or NGS errors) that contain up to 2 nucleotide substitutions were identified and associated with the appropriate intact BCs. Each genuine BC supported by only 1 read was excluded from the subsequent analysis.

5. **Identification of genuine BC-ROI combinations**. For each genuine BC, associated ROI sequences were extracted from reads carrying all BC variants and counted. A ROI sequence found in more than one half of the reads was considered as a genuine ROI. ROI sequences differing by 1 nucleotide from the genuine ROI were considered as its variations arisen due

to PCR and/or NGS errors, whereas all other ROI sequences were considered to be a result of chimeric PCR (for details, see **Additional file 3**). Only BCs coupled to genuine ROIs were counted in the subsequent calculations.

6. **Calculation of proportion of chimeric BC-ROI combinations.** First, for each genuine barcode, the proportion of reads with chimeric ROI sequences was calculated (for details, see **Additional file 3**). Next, the values obtained for all genuine BCs were averaged to get a single characteristic value for each experimental replicate.

References

1. Akhtar W, de Jong J, Pindyurin AV, Pagie L, Meuleman W, de Ridder J, Berns A, Wessels LF, van Lohuizen M, van Steensel B: **Chromatin position effects assayed by thousands of reporters integrated in parallel**. *Cell* 2013, **154**(4):914-927.
