# Supplementary material for: Optimized PCR conditions minimizing the formation of chimeric DNA molecules from MPRA plasmid libraries
Source: BMC Genomics. 2019 Jul 11;20(Suppl 7):536. doi: 10.1186/s12864-019-5847-2 (PMC6620194; doi:10.1186/s12864-019-5847-2)
Supplement: Supplementary file 3 — Table S1. Examples of identified BC–ROI combinations. (PDF 93 kb) [file 12864_2019_5847_MOESM3_ESM.pdf]

Table S1. Examples of identified BC–ROI combinations.

BCs			BC–ROI combinations			
No.	Sequence	Read count	BC sequence	ROI sequence	Read count	Proportion of chimeric molecules
1	**ATATAGTCATGCCCTAAG**	248	**ATATAGTCATGCCCTAAG**	**TAACTGAT**	242	1/251 (0.4%)
	ATATAGTCAGCCCCTAAG	3		TAACTGCT	2	
				TAACTGGT	1	
				TAACGGAT	1	
				TACCTGAT	1	
				ACTGTTGG	1	
			ATATAGTCAGCCCCTAAG	TAACTGAT	3	
2	**CTAGTATGATACACATCT**	99	**CTAGTATGATACACATCT**	**TAACTGAT**	96	0/101 (0%)
	CTAGTATGATATACATCT	2		TAACTCAT	2	
				TAACTGGT	1	
			CTAGTATGATATACATCT	TAACTGAT	2	

Sequences of genuine BCs and ROIs (i.e. the most represented sequences within the groups defined by the algorithm used) are in bold characters.
Mutant variants of genuine BCs and ROIs (BCs with up to 2 nucleotide substitutions and ROIs with 1 nucleotide substitution) are in regular font; the substitutions are underlined.
A chimeric ROI is in red.
